# *ASXL1* c.1934dup;p.Gly646Trpfs*12—a true somatic alteration requiring a new approach

**DOI:** 10.1038/s41408-017-0025-8

**Published:** 2017-12-20

**Authors:** Costas K. Yannakou, Kate Jones, Michelle McBean, Ella R. Thompson, Georgina L. Ryland, Ken Doig, John Markham, David Westerman, Piers Blombery

**Affiliations:** 1 Peter MacCallum Cancer Centre, Melbourne, VIC 3000 Australia; 2 University of Melbourne, Parkville, VIC 3010 Australia

The *additional sex combs-like 1* (*ASXL1*) gene has a central role in the epigenetic regulation of chromatin remodelling and subsequent gene transcription via multiple mechanisms. These include the regulation of histone H2A deubiquitination^1^ as well as polycomb group repressor complex 2 mediated homeobox (*HOX*) gene transcription^2^. *ASXL1* mutations are a recurrent finding in myeloid malignancies, where they are typically heterozygous in keeping with a haploinsufficiency effect^3^. Mutated *ASXL1* status has been associated with an inferior overall survival in acute myeloid leukaemia (AML)^4^, myelodysplastic syndromes (MDS)^5^, chronic myelomonocytic leukaemia (CMML)^6^, myelofibrosis^7^, aplastic anaemia^8^ and age-related clonal haematopoiesis^9^.

The majority of *ASXL1* exon 12 mutations are frameshift or nonsense and result in a C-terminal truncation of the resulting gene product. Missense mutations are also detected but these appear not to have an effect on clinical outcome and are of uncertain significance^5,6^. The most commonly detected *ASXL1* mutation is *ASXL1* NM_015338.5:c.1934dup;p.Gly646Trpfs*12 (*ASXL1* c.1934dupG), accounting for approximately half of somatic truncating mutations^4–7^. This duplication of a single guanine occurs within an eight base-pair mononucleotide guanine repeat sequence (8G repeat) that extends from c.1927 to c.1934.

Areas of repetitive sequence may be prone to accelerated mutagenesis due to replication slippage^10^. This occurs when DNA polymerase pauses and dissociates from repeated areas of sequence allowing the terminal portion of the newly synthesised strand to anneal to a different yet still complimentary location on the template. Resumption of DNA replication completes the slippage event, which may result in duplications or deletions. This process, however, has also been described as a source of polymerase chain reaction (PCR) sequencing artefact^11^. This fact, coupled with the detection by Sanger sequencing and mass spectrometry of *ASXL1* c.1934dupG within the buccal DNA of individuals with myeloid malignancies and by Sanger sequencing in the granulocyte DNA of those without, has led some to assert that this variant is not a real somatic alteration^12^. In addition, *ASXL1* c.1934dupG has been reported at a frequency of between 0.001634% (Exome Aggregation Consortium) and 2.58% (Exome Sequencing Project) in the general population by whole-exome sequencing. Despite the fact that *ASXL1* may be mutated in otherwise well individuals with age-related clonal haematopoiesis^9^, these detection frequencies may be overestimated due to artefact-related false-positive *ASXL1* c.1934dupG detection.

Various evidences in support of *ASXL1* c.1934dupG being a true somatic alteration have been put forward. These include an inability to reproduce *ASXL1* c.1934dupG detection consistently in samples known not to contain a myeloid malignancy (likely due to the use of high fidelity polymerases)^4,5,13^ and a failure to differentiate patients harbouring *ASXL1* c.1934dupG and those with other truncating ASXL1 mutations by clinical outcome^14^ or gene expression profiling^13^. However, these lines of evidence either rely on sequencing of the *ASXL1* 8G repeat or are circumstantial in nature.

We aimed to evaluate the performance of various methodologies for the detection of *ASXL1* c.1934dupG and to assess whether it is a true somatic alteration utilising a mutation-specific assay.

A cohort of 186 patients with myeloid malignancies who had blood or bone marrow samples referred for routine testing was identified from institutional databases: MDS/CMML (*n* = 47), myeloproliferative neoplasms (*n* = 81) and normal karyotype AML (*n* = 58). Sanger sequencing was performed on the entire cohort using a high fidelity DNA polymerase (Supplementary Methods). *ASXL1* c.1934dupG was detected in 14.11% (23/163) of samples (Supplementary Table 1). Visual inspection of Sanger sequencing traces revealed no evidence of slippage artefact resulting from the mononucleotide guanine repeat sequence.

As *ASXL1* c.1934dupG represents a single base-pair increase in DNA length (+1 bp), we developed a fragment analysis assay for its detection, which we applied to the entire cohort (Supplementary Methods). A +1 bp was detected by fragment analysis in all *ASXL1* c.1934dupG containing samples identified by Sanger sequencing (Supplementary Table 1). Of note, 14.81% (4/27) of +1 bp fragment analysis calls were accounted for by single base-pair duplications other than *ASXL1* c.1934dupG, demonstrating the suboptimal specificity of fragment analysis if used without correlative sequencing for *ASXL1* c.1934dupG detection.

Amplicon-based massively parallel sequencing (MPS) was performed on the entire cohort using the 26 gene Peter MacCallum Cancer Centre myeloid amplicon panel (PMCC-MAP) (Supplementary Methods). This assay uses the Fluidigm Access Array System (Fluidigm, San Francisco, CA, USA) with subsequent sequencing performed on an Illumina MiSeq sequencer (Illumina, San Diego, CA, USA).

Data generated using our institutional clinical bioinformatic pipeline (non-global amplicon alignment based on a modified Smith-Waterman algorithm (Primal) and variant calling with Varscan 2)^15^ demonstrated recurrent artefact within the 8G repeat resulting in the calling of *ASXL1* c.1934dupG at a variant allele fraction (VAF) of ≥ 3% in 44.17% (72/163) of samples known to be negative by Sanger sequencing and fragment analysis (median VAF 3.45%, VAF range 3.01%–4.87%) (Supplementary Table 1). Errors occurring within the 8G repeat were concordant between paired reads, implying the contribution of PCR to artefact generation with the PMCC-MAP (data not shown).

Mean coverage at the site of the mononucleotide guanine repeat sequence was 1039.51 paired reads per sample (4.30% of samples < 600 paired reads). *ASXL1* c.1934dupG calls from reference NA12878 DNA (Coriell Cell Repositories, Camden, NJ, USA) tested on each panel over 75 runs excluded significant inter-assay variability (data not shown).

*ASXL1* c.1934dupG VAFs were higher among the samples known to be positive by Sanger sequencing (VAF ≥ 3% in 82.60% (19/23) of samples, median VAF 7.85%, VAF range 3.68%–17.60%) and correlated positively with quantification by fragment analysis peak height ratio. Optimal sensitivity (86.96%) and specificity (93.87%) occurred at a VAF threshold of ≥ 5%, which we deem to be insufficiently discriminatory for the confident categorisation of patient samples (Supplementary Fig. 1).

Similar performance limitations concerning *ASXL1* c.1934dupG detection have been reported with the Illumina TruSight Myeloid Sequencing Panel^16^. Difficulties in accurately resolving mononucleotide repeat regions have been described with a variety of MPS technologies^17,18^ and may potentially arise from PCR, sequencing or bioinformatic sources. Substitution of the routine bioinformatics pipeline with a variant caller that utilises non-global alignment (Canary) did not significantly improve the performance of the PMCC-MAP (Supplementary Fig. 1).

In order to demonstrate definitively that *ASXL1* c.1934dupG is a true somatic alteration we developed a quantitative real-time PCR (qRT-PCR) assay (Supplementary Methods). Oligonucleotides complementary to and spanning both the *ASXL1* nine base-pair mononucleotide guanine repeat (9G repeat) (9G primer—5′-ATCGGAGGGGGGGGGT-3′) and the 8G repeat (8G primer—5′-ATCGGAGGGGGGGGT-3′) were designed and utilised in this assay together with a shared reverse primer (Fig. 1).

**Fig. 1:**
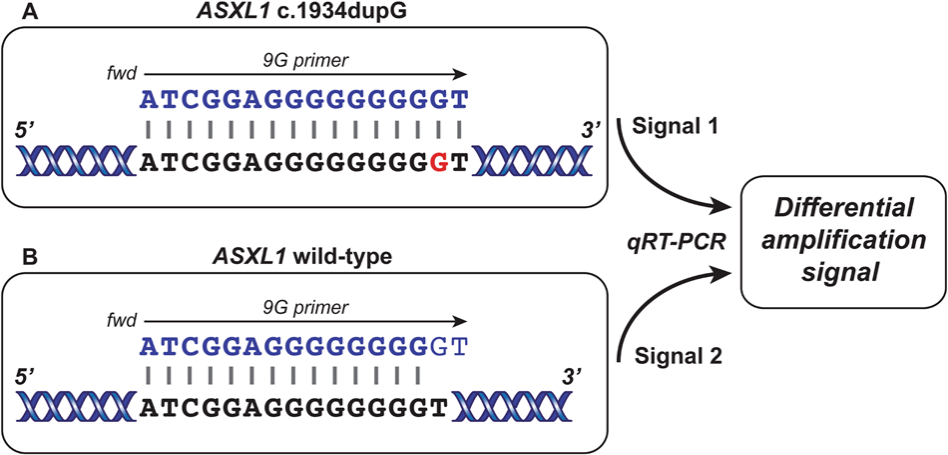
Mutation-specific mechanism of *ASXL1* c.1934dupG detection using the 9G primer **A** *ASXL1* c.1934dupG (9G repeat)—primer and template complementary. **B** Wild-type (8G repeat)—primer and template partially mismatched. Resulting PCR product amplification characteristics constitute signal

*ASXL1* c.1934dupG was detected as a heterozygous mutation within the Kasumi-1 cell line by Sanger sequencing and fragment analysis. The 9G primers affected amplification at an earlier cycle threshold (Ct) with Kasumi-1 DNA vs. *ASXL1* wild-type DNA (Fig. 2). This demonstrated the differential annealing capacity of the 9G primer for the *ASXL1* 9G and 8G repeats, providing direct and definitive proof that *ASXL1* c.1934dupG is a true somatic alteration without sequencing the mononucleotide guanine repeat sequence. Consistent with this observation, the 8G primers affected amplification at an earlier Ct with *ASXL1* wild-type DNA vs. Kasumi-1 DNA. The differential annealing capacity was less with the 8G primer vs. the 9G primer, in keeping with the presence of the 8G repeat within both Kasumi-1 DNA (50% 8G repeat—*ASXL1* c.1934dupG heterozygous) and *ASXL1* wild-type DNA (100% 8G repeat).

**Fig. 2:**
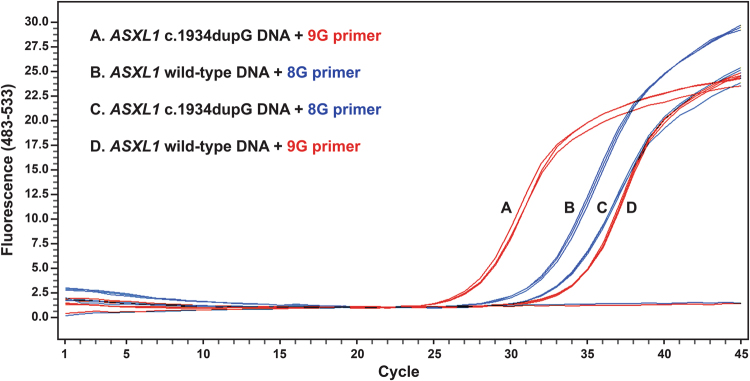
Amplification dynamics of the 9G and 8G primers with Kasumi-1 DNA (50% 9G repeat—*ASXL1* c.1934dupGheterozygous) and with *ASXL1* wild-type DNA (0% 9G repeat) A+D 9G primers—amplify Kasumi-1 DNA more efficiently than *ASXL1* wild-type DNA, greater Ct difference between DNA types due to absence of the 9G repeat within the *ASXL1* wild-type DNA. B+C 8G primers—amplify *ASXL1* wild-type DNA more efficiently than Kasumi-1 DNA, lesser Ct difference between DNA types due to presence of the 8G repeat within the Kasumi-1 DNA

In order to test for *ASXL1* c.1934dupG, the amplification dynamics of the 9G primers in relation to reference primers (Ref primers) targeting a separate region of *ASXL1* exon 12 were utilised in a novel qRT-PCR assay (Supplementary Methods). The Kasumi-1 cell line is known to be diploid for chromosome 20 by conventional karyotype^19^ and single-nucleotide polymorphism array based copy number analysis (http://www.ebi.ac.uk/arrayexpress/ (Acc. E-MTAB-4950)) indicating an *ASXL1* c.1934dupG mutation burden of 50% for Kasumi-1 DNA.

qRT-PCR was validated for use at a DNA input amount of 10 ng (linear range = 1.5625 ng–50 ng) for the detection of *ASXL1* c.1934dupG at a mutation burden of ≥ 3% utilising 2^−ΔΔCt^ analysis (Supplementary Fig. 2, Supplementary Tables 2 and 3)^20^. This level of detection is superior to that of Sanger sequencing and fragment analysis, which were both unable to detect *ASXL1* c.1934dupG below a mutation burden of 12.5% (data not shown). This method can be modified for the purposes of absolute quantification of *ASXL1* c.1934dupG mutation burden through the use of a reference curve derived from serial dilutions of Kasumi-1 DNA into wild-type DNA (Supplementary Fig. 2).

qRT-PCR detected *ASXL1* c.1934dupG within each of 15 patient samples known to be positive by Sanger sequencing (Supplementary Table 4). In addition, the value of the improved level of detection afforded by qRT-PCR has been illustrated in a number of clinical cases of myeloid malignancy (Supplementary Table 5). Such clinical contexts include the detection of otherwise undetectable *ASXL1* c.1934dupG containing subclones at diagnosis and the monitoring of their responses to cytotoxic therapy, as well as the monitoring of measurable residual disease after allogeneic stem cell transplantation.

In summary, we have definitively proven that *ASXL1* c.1934dupG is a true somatic alteration. Due to the suboptimal ability of MPS panels to sequence the mononucleotide guanine repeat in which *ASXL1* c.1934dupG occurs, the use of this technology in isolation is associated with false-negative and artefact-related false-positive results. This is of significant clinical relevance due to the prevalence of truncating *ASXL1* mutations and their effect on clinical outcome in patients with myeloid malignancies. For this reason we recommend the synchronous use of a sensitive, adjunctive method to ensure the comprehensive detection of all clinically relevant *ASXL1* mutations in this patient population. The qRT-PCR assay described herein represents a novel method of *ASXL1* c.1934dupG detection, the greater sensitivity of which may add value in certain clinical contexts.

## Supplementary information

Supplementary Methods
Supplementary Figure 1
Supplementary Figure 2
Supplementary Figure Legends
Supplementary Table 1
Supplementary Table 2
Supplementary Table 3
Supplementary Table 4
Supplementary Table 5
Supplementary Table Legends

## References

[1] Sahtoe DD, van Dijk WJ, Ekkebus R, Ovaa H, Sixma TK (2016). BAP1/ASXL1 recruitment and activation for H2A deubiquitination. Nat. Commun..

[2] Abdel-Wahab O (2012). ASXL1 mutations promote myeloid transformation through loss of PRC2-mediated gene repression. Cancer Cell..

[3] Gelsi-Boyer V (2012). Mutations in ASXL1 are associated with poor prognosis across the spectrum of malignant myeloid diseases. J. Hematol. Oncol..

[4] Schnittger S (2013). ASXL1 exon 12 mutations are frequent in AML with intermediate risk karyotype and are independently associated with an adverse outcome. Leukemia.

[5] Thol F (2011). Prognostic significance of ASXL1 mutations in patients with myelodysplastic syndromes. J. Clin. Oncol..

[6] Patnaik MM (2014). ASXL1 and SETBP1 mutations and their prognostic contribution in chronic myelomonocytic leukemia: a two-center study of 466 patients. Leukemia.

[7] Vannucchi AM (2013). Mutations and prognosis in primary myelofibrosis. Leukemia.

[8] Yoshizato T (2015). Somatic mutations and clonal hematopoiesis in aplastic anemia. N. Engl. J. Med..

[9] Jaiswal S (2014). Age-related clonal hematopoiesis associated with adverse outcomes. N. Engl. J. Med..

[10] Viguera E, Canceill D, Ehrlich SD (2001). Replication slippage involves DNA polymerase pausing and dissociation. Embo. J..

[11] Fazekas A, Steeves R, Newmaster S (2010). Improving sequencing quality from PCR products containing long mononucleotide repeats. Biotechniques.

[12] Abdel-Wahab O, Kilpivaara O, Patel J, Busque L, Levine RL (2010). The most commonly reported variant in ASXL1 (c.1934dupG;p.Gly646TrpfsX12) is not a somatic alteration. Leukemia.

[13] Metzeler KH (2011). ASXL1 mutations identify a high-risk subgroup of older patients with primary cytogenetically normal AML within the ELN Favorable genetic category. Blood.

[14] Itzykson R (2013). Prognostic score including gene mutations in chronic myelomonocytic leukemia. J. Clin. Oncol..

[15] Koboldt DC (2012). VarScan 2: somatic mutation and copy number alteration discovery in cancer by exome sequencing. Genome Res..

[16] Thomas M (2017). Integration of technical, bioinformatic, and variant assessment approaches in the validation of a targeted next-generation sequencing panel for myeloid malignancies. Arch. Pathol. Lab. Med..

[17] Quail MA (2012). A tale of three next generation sequencing platforms: comparison of Ion Torrent, Pacific Biosciences and Illumina MiSeq sequencers. BMC Genom..

[18] Goodwin S, McPherson JD, McCombie WR (2016). Coming of age: ten years of next-generation sequencing technologies. Nat. Rev. Genet..

[19] Asou H (1991). Establishment of a human acute myeloid leukemia cell line (Kasumi-1) with 8;21 chromosome translocation. Blood.

[20] Livak KJ, Schmittgen TD (2001). Analysis of relative gene expression data using real-time quantitative PCR and the 2(-Delta Delta C(T)) Method. Methods.

