# Supplementary material for: ASXL1 c.1934dup;p.Gly646Trpfs*12—a true somatic alteration requiring a new approach
Source: Blood Cancer J. 2017 Dec 20;7(12):656. doi: 10.1038/s41408-017-0025-8 (PMC5802455; doi:10.1038/s41408-017-0025-8)
Supplement: Supplementary file 1 — Supplementary Methods [file 41408_2017_25_MOESM1_ESM.docx]

**Supplementary Methods**

Patient samples

Samples were selected from stored routine diagnostic cases identified on institutional databases from patients with myelodysplastic syndrome (MDS), chronic myelomonocytic leukaemia (CMML), myeloproliferative neoplasms (MPNs) and normal karyotype acute myeloid leukaemia (AML) as defined by WHO 2008 diagnostic criteria.

DNA from peripheral blood and bone marrow aspirate samples was extracted using the EZ1 DNA Tissue Kit (Qiagen, Hilden, Germany) on the EZ1 Advanced XL system (Qiagen) as per the manufacturer’s instructions. DNA quantification was performed using the Qubit dsDNA BR Assay Kit (Thermo Fisher Scientific, Waltham, MA, USA) using the Qubit 2.0 Fluorometer (Thermo Fisher Scientific).

Cell culture

The myeloblast cell line Kasumi-1 (American Type Culture Collection, Manassas, VA, USA) was cultured in Roswell Park Memorial Institute (RPMI) 1640 medium (Thermo Fisher Scientific) supplemented with 10% vol/vol fetal bovine serum (Sigma-Aldrich, St. Louis, MI, USA) and 100 U/ml of penicillin/streptomycin (Invitrogen, Carlsbad, CA, USA).

Sanger sequencing

A region of *ASXL1* exon 12 that contains the eight base pair mononucleotide guanine repeat was amplified by PCR on an ABI Veriti Thermal Cycler (Thermo Fisher Scientific) using the M13 tailed *ASXL1* specific primers Forward ( 5’ tgtaaaacgacggccagtTGCCATGACCCTTAAGCTACT 3’) and Reverse (5’ caggaaacagctatgaccAGCTCTGGACATGGCAGTTC 3’). Each PCR reaction contained 1X Amplitaq Gold 360 Master Mix (Thermo Fisher Scientific), 400 nM each primer and 10 ng of input DNA in a 10 μl reaction volume. PCR conditions were an initial denaturation at 95°C for 5 minutes followed by 40 rounds of cycling at 94°C for 30 seconds, 60°C for 40 seconds and 72°C for 45 seconds.

The PCR reaction was purified using the Agencourt AMPure XP system (Beckman Coulter, Brea, CA, USA). Sequencing was performed using M13 forward and reverse primers and the BigDye Terminator v3.1 Cycle Sequencing Kit (Thermo Fisher Scientific). The sequencing reaction was then purified using the AxyPrep MAG DyeClean Kit (Fisher Biotec, Wembley, WA, Australia) and analysed by capillary electrophoresis on an ABI 3730 DNA Analyser (Thermo Fisher Scientific). Sequencing data was analysed using Mutation Surveyor v4.0.5 software (SoftGenetics, State College, PA, USA).

Fragment analysis

A region of *ASXL1* exon 12 that contains the eight base pair mononucleotide guanine repeat was amplified by PCR on an ABI Veriti Thermal Cycler (Thermo Fisher Scientific) using the *ASXL1* specific primers Forward (5’ acactgacgacatggttctacaACCCTGGGTGGTTAAAGGTC 3’) and Reverse (5’ HEX-GTTCCGGCCTGGGTATGCTC 3’). Each PCR reaction contained 1X PCR Buffer (200 mM Tris-HCl (pH 8.4) and 500 mM KCl), 1.5 mM MgCl_2_, 200nM each dNTP, 125 nM each primer, 2 U Invitrogen Platinum Taq DNA Polymerase (Thermo Fisher Scientific) and 25 ng of input DNA in a 10 μl reaction volume. PCR conditions were an initial denaturation at 95°C for 7 min followed by 35 rounds of cycling at 95°C for 45 seconds, 60°C for 45 seconds and 72°C for 90 seconds before a final extension at 72°C for 10 minutes.

The resulting PCR product was combined with HI-DI formamide (Thermo Fisher Scientific) and GeneScan 500 ROX dye size standard (Thermo Fisher Scientific) and heat denatured at 95°C for 5 minutes. Samples were sized by capillary electrophoresis on an ABI 3730 DNA Analyser (Thermo Fisher Scientific). Fragment analysis data was analysed using GeneMarker v1.97 software (SoftGenetics, State College, PA, USA).

Massively parallel sequencing – Peter MacCallum Cancer Centre myeloid amplicon panel

Library preparation was performed using the Fluidigm Access Array System (Fluidigm, San Francisco, CA, USA). PCR amplification occurred within an Access Array 48.48 integrated fluidic circuit (IFC) using 50 ng of input DNA. PCR products were indexed using sample-specific barcode primers (Fluidigm) as per the manufacturer’s instructions. Samples were pooled and the resulting library was purified using the Agencourt AMPure XP system (Beckman Coulter) and quantified on a 2200 TapeStation instrument (Agilent Technologies, Santa Clara, CA, USA). Libraries were then denatured and diluted as per the manufacturer’s instructions and 150 base pair paired-end sequencing was performed on an Illumina MiSeq sequencer (Illumina, San Diego, CA, USA) using MiSeq v2 chemistry.

Sequencing data was then de-multiplexed and FASTQ files were generated by CASAVA v1.8.2. Reads were assembled and aligned using Primal (an in-house developed non-global alignment method based on a modified Smith-Waterman algorithm) and variants called using VarScan 2. An in-house developed combination non-global alignment method and variant caller known as Canary was used as an alternative to Primal and Varscan 2 only where specifically indicated in the text. Both of these methods discard variants with coverage of <100 total reads or <20 mutant reads.

*ASXL1* c.1934dupG was annotated and reported using in-house-developed software (PathOS) that integrates variant annotation with sequencing characteristics as well as facilitating variant curation and report generation.

The amplicon that covers the eight base pair mononucleotide guanine repeat is generated using the CS1/CS2 tagged primers Forward (5’ acactgacgacatggttctacaGTGCTCTGCAGGTCCGAG 3’) and Reverse (5’ tacggtagcagagacttggtctACGTACACTTTCCAGGGGTG 3’).

Quantitative real-time PCR (qRT-PCR)

Quantification of *ASXL1* c.1934dupG mutation burden was performed using qRT-PCR performed on a LightCycler 480 (Roche, Basel, Switzerland). Each PCR reaction contained 1X Amplitaq Gold 360 Master Mix (Thermo Fisher Scientific), 200 nM each primer (see below), 5 µM Syto9 (Thermo Fisher Scientific) and 10 ng of input DNA in a 10 μl reaction volume.

| Primer | Target | Forward | Reverse |
| --- | --- | --- | --- |
| 8G | *ASXL1* wild type | 5’ ATCGGAGGGGGGGGT 3’ | 5’ AGCTCTGGACATGGCAGTTC 3’ |
| 9G | *ASXL1* c.1934dupG | 5’ ATCGGAGGGGGGGGGT 3’ | 5’ AGCTCTGGACATGGCAGTTC 3’ |
| Ref | *ASXL1* exon 12 | 5’ acactgacgacatggttctaca  AGCCTCTGGAGCCTTCTTCT 3’ | 5’ tacggtagcagagacttggtct  AAGGAAAGTGATGCACTGTGG 3’ |

PCR conditions were an initial denaturation at 95°C for 5 minutes followed by 45 rounds of cycling at 94°C for 30 seconds, 60°C for 45 seconds and 72°C for 45 seconds. qRT-PCR data was analysed using LightCycler 480 v1.5.0 software (Roche). Cycle thresholds (Cts) were set at 3.55 RFU for the 9G primer and 7.05 RFU for the Ref primer.

Serial dilution experiments with reducing amounts of wild type DNA were performed using 9G and Ref primers at DNA inputs spanning 0.78125 ng – 100 ng (Supplementary Figure 2A, Supplementary Figure 2B). Serial dilution experiments of Kasumi-1 DNA into wild type DNA (10 ng and 50 ng input amounts) were performed using 9G primers at *ASXL1* c.1934dupG mutation burdens spanning 0.390625% – 50% (Supplementary Figure 3B, Supplementary Figure 3C).

All samples were run in duplicate at minimum alongside both a high positive (Kasumi-1 DNA – 50% *ASXL1* c.1934dupG mutation burden), a low positive (Kasumi-1 DNA – 3% *ASXL1* c.1934dupG mutation burden) and a wild type control. Replicates were averaged prior to analysis. *ASXL1* c.1934dupG (at 3% mutation burden or greater) was considered detected if fold change between the wild type sample and the sample of interest (FC (WT-Sample)) exceeded the -95% confidence limit (one-tailed) of the mean fold change between wild type DNA and the low positive control (FC (WT-3%)) over six experiments (1.39) and if FC (WT-Sample) exceeded FC (WT-3%) on the relevant experiment (Supplementary Table 2).
