# Supplementary figures and images for: ASXL1 c.1934dup;p.Gly646Trpfs*12—a true somatic alteration requiring a new approach
Source: Blood Cancer J. 2017 Dec 20;7(12):656. doi: 10.1038/s41408-017-0025-8 (PMC5802455; doi:10.1038/s41408-017-0025-8)

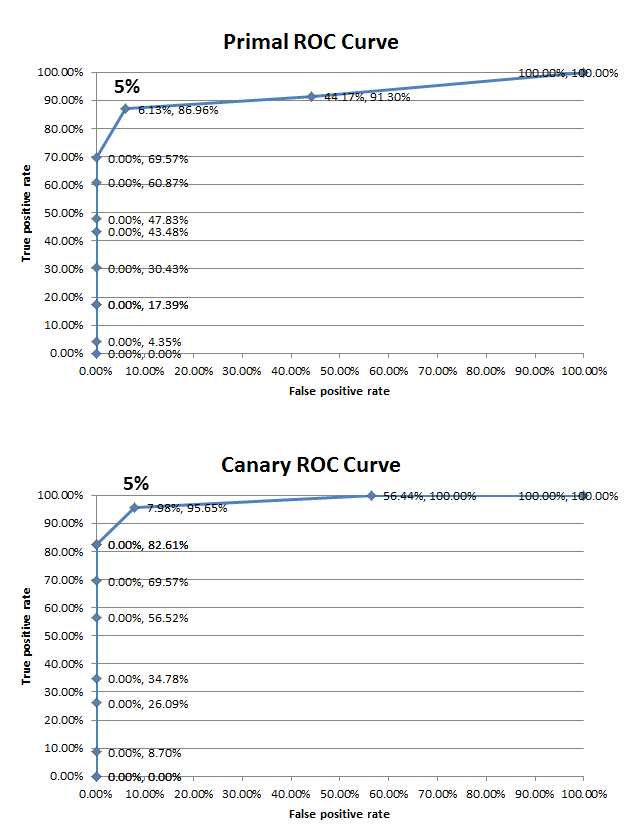

Supplement: Supplementary file 2 — Supplementary Figure 1 [file 41408_2017_25_MOESM2_ESM.tif]

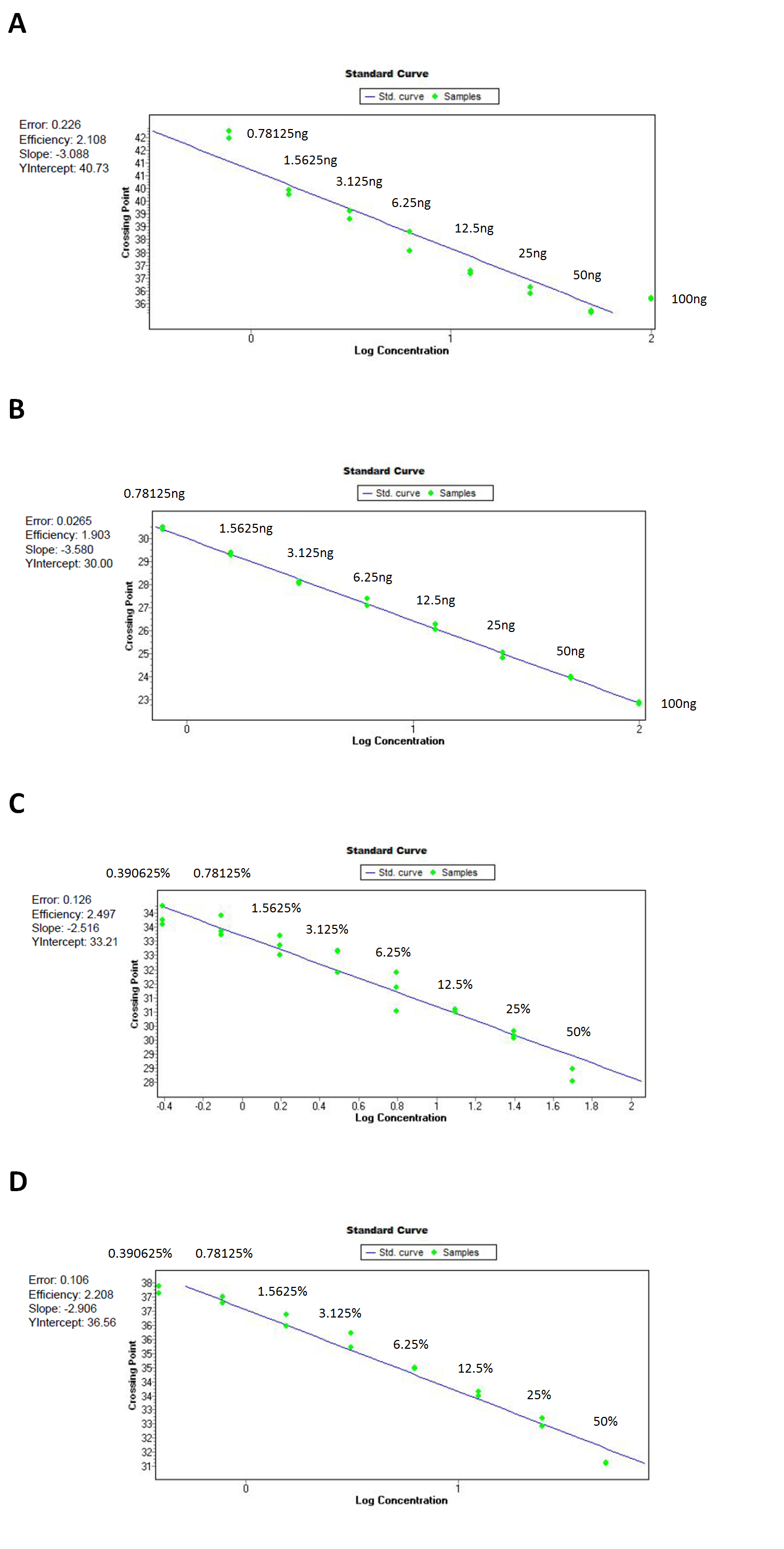

Supplement: Supplementary file 3 — Supplementary Figure 2 [file 41408_2017_25_MOESM3_ESM.tif]
