# Supplementary material for: ASXL1 c.1934dup;p.Gly646Trpfs*12—a true somatic alteration requiring a new approach
Source: Blood Cancer J. 2017 Dec 20;7(12):656. doi: 10.1038/s41408-017-0025-8 (PMC5802455; doi:10.1038/s41408-017-0025-8)
Supplement: Supplementary file 4 — Supplementary Figure Legends [file 41408_2017_25_MOESM4_ESM.docx]

**Supplementary Figure Legends**

Supplementary Figure 1

Receiver operator characteristic curves of *ASXL1* c.1934dupG VAFs (Primal and Canary – highest VAF per sample) demonstrating the optimal detection threshold to be 5%.

Supplementary Figure 2

Serial dilution curves demonstrating performance characteristics of the qRT-PCR assay:

- A – 9G primers tested with various amounts of wild-type DNA:
  - Linear range = 1.5625 ng – 50 ng
- B – Ref primers tested with various amounts of wild-type DNA:
  - Linear range = 0.78125 ng – 100 ng
- C & D – 9G primers tested with various *ASXL1* c.1934dupG mutation burdens (serial dilutions of Kasumi-1 DNA):
  - C – Kasumi-1 DNA at 50ng input:
    - Linear range = 0.78125% – 25%
  - D – Kasumi-1 DNA at 10ng input:
    - Linear range = 0.78125% – 25%
