# Supplementary material for: ASXL1 c.1934dup;p.Gly646Trpfs*12—a true somatic alteration requiring a new approach
Source: Blood Cancer J. 2017 Dec 20;7(12):656. doi: 10.1038/s41408-017-0025-8 (PMC5802455; doi:10.1038/s41408-017-0025-8)
Supplement: Supplementary file 6 — Supplementary Table 2 [file 41408_2017_25_MOESM6_ESM.docx]

**Supplementary Table 2:**

|  | 9G WT Ct | Ref WT Ct | ΔCt^*^ WT |  | 9G 3% Ct | Ref 3% Ct | ΔCt^*^ 3% |  | ΔΔCt (WT-3%)^†^ |  | FC (WT-3%)^‡^ |
| --- | --- | --- | --- | --- | --- | --- | --- | --- | --- | --- | --- |
| Experiment A | 37.83 | 26.07 | 11.76 |  | 35.56 | 26.46 | 9.10 |  | -2.66 |  | 6.32 |
| Experiment B | 38.07 | 25.98 | 12.09 |  | 36.55 | 25.70 | 10.85 |  | -1.24 |  | 2.36 |
| Experiment C | 35.46 | 25.13 | 10.33 |  | 33.32 | 25.04 | 8.28 |  | -2.05 |  | 4.14 |
| Experiment D | 32.50 | 24.35 | 8.15 |  | 30.95 | 24.35 | 6.60 |  | -1.55 |  | 2.93 |
| Experiment E | 31.62 | 24.69 | 6.93 |  | 30.05 | 24.65 | 5.40 |  | -1.53 |  | 2.89 |
| Experiment F | 32.39 | 24.57 | 7.82 |  | 30.36 | 24.45 | 5.91 |  | -1.91 |  | 3.76 |
|  |  |  |  |  |  |  |  |  |  |  |  |
|  |  |  |  |  |  |  |  |  |  | Mean  FC (WT-3%) | 3.73 |
|  |  |  |  |  |  |  |  |  |  | SD  FC (WT-3%) | 1.42 |
|  |  |  |  |  |  |  |  |  |  | -95% CL  FC (WT-3%)^§^ | 1.39 |

9G, 9G primers; WT, wild-type; Ct, cycle threshold; Ref, reference primers; 3%, Kasumi-1 DNA – 3% *ASXL1* c.1934dupG mutation burden; FC, fold change; SD, standard deviation; CL, confidence limit

*ΔCt = 9GCt-Ref Ct

†ΔΔCt (WT-3%) = ΔCt wild-type DNA - ΔCt Kasumi-1 DNA – 3% *ASXL1* c.1934dupG mutation burden

‡FC (WT-3%) = 2^-ΔΔCt (WT-3%)^

§-95% confidence limit (one-tailed) FC (WT-3%) = mean FC (WT-3%)-(1.645xSD FC (WT-3%))
