# Supplementary material for: ASXL1 c.1934dup;p.Gly646Trpfs*12—a true somatic alteration requiring a new approach
Source: Blood Cancer J. 2017 Dec 20;7(12):656. doi: 10.1038/s41408-017-0025-8 (PMC5802455; doi:10.1038/s41408-017-0025-8)
Supplement: Supplementary file 7 — Supplementary Table 3 [file 41408_2017_25_MOESM7_ESM.docx]

**Supplementary Table 3:**

| Samples | 9G Ct | Ref Ct | ΔCt^*^ |  | ΔΔCt (WT-Sample)^†^ |  | FC (WT-Sample)^‡^ |
| --- | --- | --- | --- | --- | --- | --- | --- |
| WT | 32.39 | 24.57 | 7.82 |  | 0.00 |  | 1.00 |
| 3% | 30.36 | 24.45 | 5.91 |  | -1.91 |  | 3.76 |
|  |  |  |  |  |  |  |  |
| L1 | 32.94 | 24.29 | 8.65 |  | 0.83 |  | 0.56 |
| L2 | 32.78 | 24.06 | 8.72 |  | 0.90 |  | 0.54 |
| L3 | 32.57 | 24.52 | 8.05 |  | 0.23 |  | 0.85 |
| L4 | 32.42 | 24.55 | 7.87 |  | 0.05 |  | 0.97 |
| L5 | 31.87 | 24.18 | 7.69 |  | -0.13 |  | 1.09 |
| L6 | 32.40 | 24.42 | 7.98 |  | 0.16 |  | 0.90 |
| L7 | 32.41 | 24.39 | 8.02 |  | 0.20 |  | 0.87 |
| L8 | 33.04 | 24.16 | 8.88 |  | 1.06 |  | 0.48 |
| L9 | 33.51 | 24.44 | 9.07 |  | 1.25 |  | 0.42 |
| L10 | 32.46 | 24.39 | 8.07 |  | 0.25 |  | 0.84 |
| L11 | 32.33 | 24.51 | 7.82 |  | 0.00 |  | 1.00 |
| L12 | 31.81 | 24.34 | 7.47 |  | -0.35 |  | 1.27 |
| L13 | 32.09 | 24.47 | 7.62 |  | -0.20 |  | 1.15 |
| L14 | 32.11 | 24.30 | 7.81 |  | -0.01 |  | 1.01 |
| L15 | 32.22 | 24.33 | 7.89 |  | 0.07 |  | 0.95 |
|  |  |  |  |  |  |  |  |
|  |  |  |  |  |  | Mean  FC (WT-L) | 0.86 |
|  |  |  |  |  |  | SD  FC (WT-L) | 0.25 |
|  |  |  |  |  |  | +95% CL  FC (WT-L)^§^ | 1.28 |

9G, 9G primers; Ct, cycle threshold; Ref, reference primers; WT, wild-type; Sample, sample of interest; FC, fold change; L, L1-L15; SD, standard deviation; CL, confidence limit

*ΔCt = 9GCt-Ref Ct

†ΔΔCt (WT-Sample) = ΔCt wild-type DNA - ΔCt Sample DNA

‡FC (WT-Sample) = 2^-ΔΔCt (WT-Sample)^

§-95% confidence limit (one-tailed) FC (WT-Sample) = mean FC (WT-Sample)-(1.645xSD FC (WT-Sample))
