# Supplementary material for: ASXL1 c.1934dup;p.Gly646Trpfs*12—a true somatic alteration requiring a new approach
Source: Blood Cancer J. 2017 Dec 20;7(12):656. doi: 10.1038/s41408-017-0025-8 (PMC5802455; doi:10.1038/s41408-017-0025-8)
Supplement: Supplementary file 8 — Supplementary Table 4 [file 41408_2017_25_MOESM8_ESM.docx]

**Supplementary Table 4:**

| Patient sample | Fragment analysis PHR | VAF% - Primal^*^ | VAF% - Canary^*^ | qRT-PCR^†^ |
| --- | --- | --- | --- | --- |
| MDS 7 | 0.48 | 9.80 | 10.00 | DETECTED |
| MDS 9 | 0.56 | 11.15 | 10.40 | DETECTED |
| MDS 36 | 0.52 | 6.19 | 6.71 | DETECTED |
| MDS 47 | 0.55 | 8.95 | 9.27 | DETECTED |
| MPN 9 | 0.17 | 4.06 | 4.24 | DETECTED |
| MPN 20 | 0.12 | 3.68 | 3.44 | DETECTED |
| MPN 27 | 0.53 | 5.90 | 7.07 | DETECTED |
| MPN 42 | 0.46 | 6.79 | 6.93 | DETECTED |
| MPN 53 | 0.60 | 9.41 | 10.02 | DETECTED |
| MPN 56 | 0.33 | 7.85 | 7.38 | DETECTED |
| MPN 77 | 0.55 | 11.26 | 11.63 | DETECTED |
| AML 17 | 0.59 | 17.60 | 10.38 | DETECTED |
| AML 40 | 0.57 | 8.73 | 8.42 | DETECTED |
| AML 50 | 0.62 | 8.97 | 8.11 | DETECTED |
| AML 58 | 0.46 | 9.64 | 9.39 | DETECTED |

PHR, peak height ratio; VAF, variant allele fraction; qRT-PCR, quantitative real-time polymerase chain reaction

*Highest VAF per sample

†*ASXL1* c.1934dupG (at 3% mutation burden or greater) was considered detected if fold change between the wild-type sample and the sample of interest (FC (WT-Sample)) exceeded the -95% confidence limit (one-tailed) of the mean fold change between wild-type DNA and Kasumi-1 DNA – 3% *ASXL1* c.1934dupG mutation burden (FC (WT-3%)) over six experiments (1.39) and if FC (WT-Sample) exceeded FC (WT-3%) on the relevant experiment (Supplementary Table 2)
