# Supplementary material for: ASXL1 c.1934dup;p.Gly646Trpfs*12—a true somatic alteration requiring a new approach
Source: Blood Cancer J. 2017 Dec 20;7(12):656. doi: 10.1038/s41408-017-0025-8 (PMC5802455; doi:10.1038/s41408-017-0025-8)
Supplement: Supplementary file 9 — Supplementary Table 5 [file 41408_2017_25_MOESM9_ESM.docx]

**Supplementary Table 5:**

|  | Myeloid malignancy | Time point | Method of *ASXL1* c.1934dupG detection | | *ASXL1* exon 12 |
| --- | --- | --- | --- | --- | --- |
|  |  |  | qRT-PCR  (FC (WT-Sample))* | Fragment analysis | Massively parallel sequencing |
| Case 1 | MPN | Diagnosis | DETECTED (5.43) | NOT DETECTED | NOT DETECTED |
| Case 2 | AML (transformed MF) | Post remission induction chemotherapy | DETECTED (12.17) | NOT DETECTED | NOT DETECTED |
| Case 3 | MDS | Diagnosis | DETECTED (2.91) | NOT DETECTED | NOT DETECTED |
|  |  | 2 weeks post azacitidine | NOT  DETECTED (1.89) |  |  |
|  |  | 9 weeks post azacitidine | NOT DETECTED (0.84) |  |  |
| Case 4 | AML (transformed MDS/MPN) | Diagnosis | DETECTED (4.18) | NOT DETECTED | NOT DETECTED |
|  |  | 3 weeks post azacitidine/  venetoclax | NOT DETECTED (1.31) |  |  |
|  | AML in complete remission, persisting MDS/MPN | 3 months post azacitidine/  venetoclax | NOT DETECTED (1.96) |  |  |
| Case 5 | MF | Relapse  post ASCT | DETECTED (4.79) | NOT DETECTED | NOT DETECTED |
|  |  | 6 months post supportive care alone | DETECTED  (5.86) | NOT DETECTED | NOT DETECTED |
| Case 6 | MF | 2 months  pre ASCT | DETECTED (53.35) | DETECTED | NOT DETECTED |
|  |  | 1 month  pre ASCT | DETECTED (55.43) |  |  |
|  |  | Day 35  post ASCT | NOT DETECTED (1.78) | NOT DETECTED |  |
|  |  | Day 64  post ASCT | NOT DETECTED (0.88) |  |  |
|  |  | Day 100  post ASCT | NOT DETECTED (0.69) |  |  |

qRT-PCR, quantitative real-time polymerase chain reaction; FC, fold change; WT, wild-type; Sample, sample of interest; MPN, myeloproliferative neoplasm; MF, myelofibrosis; ASCT, allogeneic stem cell transplantation; AML, acute myeloid leukaemia; MDS, myelodysplastic syndrome

†*ASXL1* c.1934dupG (at 3% mutation burden or greater) was considered detected if fold change between the wild-type sample and the sample of interest (FC (WT-Sample)) exceeded the -95% confidence limit (one-tailed) of the mean fold change between wild-type DNA and Kasumi-1 DNA – 3% *ASXL1* c.1934dupG mutation burden (FC (WT-3%)) over six experiments (1.39) and if FC (WT-Sample) exceeded FC (WT-3%) on the relevant experiment (Supplementary Table 2)
