# Supplementary material for: ASXL1 c.1934dup;p.Gly646Trpfs*12—a true somatic alteration requiring a new approach
Source: Blood Cancer J. 2017 Dec 20;7(12):656. doi: 10.1038/s41408-017-0025-8 (PMC5802455; doi:10.1038/s41408-017-0025-8)
Supplement: Supplementary file 10 — Supplementary Table Legends [file 41408_2017_25_MOESM10_ESM.docx]

**Supplementary Table Legends**

Supplementary Table 1

Cohort of 186 patients with myeloid malignancies tested for *ASXL1* c.1934dupG by massively parallel sequencing, Sanger sequencing and fragment analysis.

Supplementary Table 2

Validation data for quantitative real-time PCR for *ASXL1* c.1934dupG detection at 3% mutation burden:

- 3% *ASXL1* c.1934dupG mutation burden detection threshold was set at the -95% confidence limit (one-tailed) of the mean fold change between wild-type DNA and Kasumi-1 DNA – 3% *ASXL1* c.1934dupG mutation burden (FC (WT-3%)) over six experiments (1.39)
- *ASXL1* c.1934dupG (at 3% mutation burden or greater) was considered detected if FC between the wild-type sample and the sample of interest (FC (WT-Sample)) exceeded 1.39 and if FC (WT-Sample) exceeded FC (WT-3%) on the relevant experiment

Supplementary Table 3

Experiment F – quantitative real-time PCR results for patient samples containing various B-cell lymphoid malignancies (L1 – L15) in which *ASXL1* mutations are not recurrent:

- +95% confidence limit (one-tailed) of the mean fold change between wild-type DNA and each patient sample (1.28) was less than the -95% confidence limit (one-tailed) of the mean fold change between wild-type DNA and Kasumi-1 DNA – 3% *ASXL1* c.1934dupG mutation burden (FC (WT-3%)) over six experiments (1.39) (Supplementary Table 2) and less than FC (WT-3%)) on this experiment, validating the absence of *ASXL1* c.1934dupG within the wild-type sample used in this and prior experiments

Supplementary Table 4

Massively parallel sequencing, fragment analysis and quantitative real-time PCR results for patient samples known to be positive for *ASXL1* c.1934dupG by Sanger sequencing.

Supplementary Table 5

Clinical cases illustrating the use of quantitative real-time PCR for the detection of otherwise undetectable *ASXL1* c.1934dupG containing subclones at diagnosis and the monitoring of their response to cytotoxic therapy, as well for as the monitoring of measurable residual disease after allogeneic stem cell transplantation.
